# Systematic review of economic evaluations of human cell-derived wound care products for the treatment of venous leg and diabetic foot ulcers

**DOI:** 10.1186/1472-6963-9-115

**Published:** 2009-07-10

**Authors:** Astrid Langer, Wolf Rogowski

**Affiliations:** 1 Institute of Health Economics and Health Care Management, Munich School of Management, Ludwig-Maximilians-Universität München, Munich, Germany; 2 Institute of Health Economics and Health Care Management, Helmholtz Zentrum München, German Research Center for Environmental Health, Neuherberg, Germany

## Abstract

**Background:**

Tissue engineering is an emerging field. Novel bioengineered skin substitutes and genetically derived growth factors offer innovative approaches to reduce the burden of diabetic foot and venous leg ulcers for both patients and health care systems. However, they frequently are very costly. Based on a systematic review of the literature, this study assesses the cost-effectiveness of these growth factors and tissue-engineered artificial skin for treating chronic wounds.

**Methods:**

On the basis of an extensive explorative search, an appropriate algorithm for a systematic database search was developed. The following databases were searched: BIOSIS Previews, CRD databases, Cochrane Library, EconLit, Embase, Medline, and Web of Science. Only completed and published trial- or model-based studies which contained a full economic evaluation of growth factors and bioengineered skin substitutes for the treatment of chronic wounds were included. Two reviewers independently undertook the assessment of study quality. The relevant studies were assessed by a modified version of the Consensus on Health Economic Criteria (CHEC) list and a published checklist for evaluating model-based economic evaluations.

**Results:**

Eleven health economic evaluations were included. Three biotechnology products were identified for which topical growth factors or bioengineered skin substitutes for the treatment of chronic leg ulceration were economically assessed: (1) Apligraf^®^, a bilayered living human skin equivalent indicated for the treatment of diabetic foot and venous leg ulcers (five studies); (2) Dermagraft^®^, a human fibroblast-derived dermal substitute, which is indicated only for use in the treatment of full-thickness diabetic foot ulcers (one study); (3) REGRANEX^® ^Gel, a human platelet-derived growth factor for the treatment of deep neuropathic diabetic foot ulcers (five studies). The studies considered in this review were of varying and partly low methodological quality. They calculated that due to shorter treatment periods, fewer complications and fewer inpatient episodes the initial cost of the novel biotechnology products may be offset, making the treatment cost-effective or even cost-saving. The results of most studies were sensitive to initial costs of the products and the evidence of effectiveness.

**Conclusion:**

The study results suggest that some growth factors and tissue-engineered artificial skin products feature favourable cost-effectiveness ratios in selected patient groups with chronic wounds. Despite the limitations of the studies considered, it is evident that health care providers and coverage decision makers should take not only the high cost of the biotechnology product but the total cost of care into account when deciding about the appropriate allocation of their financial resources. However, not only the cost-effectiveness but first of all the effectiveness of these novel biotechnology products deserve further research.

## Background

The loss or failure of a tissue or organ due to congenital abnormalities, disease, trauma, or aging is a frequent and costly problem in health care. A new discipline, tissue engineering, aims at developing biological substitutes to repair, restore or improve tissue or organ function that has been lost. It is an emerging interdisciplinary field that applies the principles of physical sciences, engineering and the life sciences. In this field, skin substitutes and growth factors offer promise in the treatment of chronic and acute wounds, burns, and various other skin disorders.

Diabetic foot and venous leg ulcers are frequent and costly complications of their underlying diseases and thus represent a critical issue for public health. The economic burden of diabetic foot ulcers can be explained by several factors [1]:

▪ Late management of patients with diabetes

▪ High recurrence and amputation rates

▪ Complexity of treatment modalities on patients with osteomyelitis

▪ High morbidity and mortality rates after amputation

Novel biotechnology products may reduce this burden for both the patient and the health care system in a cost-effective or even cost-saving way. To gain market access, manufacturers increasingly have to establish not only the efficacy of their products, but also whether these provide a cure at an accepted cost per health gain.

The objective of this systematic review was to assess the health economic evidence of bioengineered skin substitutes and growth factors for the treatment of chronic leg ulceration. The review forms part of a study on regenerative medicine in Germany, funded by the German Ministry of Education and Research.

## Methods

Only full health economic evaluations (cost-minimisation, cost-effectiveness, cost-utility or cost-benefit analyses) of topical growth factors and bioengineered skin products for the treatment of therapy-resistant chronic wounds in English, French or German language were considered. Publications outside the above categories were excluded from this review but used for reference tracking. Also, the study did not include a systematic review of effectiveness studies but only assessed those used within the identified economic evaluations. Acellular artificial skin and skin substitutes indicated only for use in burns were excluded. Furthermore, economic evaluations included in an earlier systematic review conducted by Ho et al. [2] were excluded to avoid duplication of efforts in reviewing and synthesizing evidence. On the basis of an extensive explorative search, an appropriate algorithm for a systematic database search was developed. The following databases were searched to identify relevant literature: BIOSIS Previews, Cochrane Library, Database of Abstracts of Reviews of Effects (DARE), EconLit, Embase, HTA Database, Medline, NHS Economic Evaluation Database (NHS EED), and Web of Science. Additionally, the internet was searched by Google and Google Scholar, and references of recent economic evaluations and reviews were tracked. The search was updated until and including July 2008. In order to include studies published while this review was being produced, the literature search was updated till the end of November 2008. This update was restricted to the PubMed database, which contains Medline. The exploratory search generated a set of eleven economic evaluations of cell-derived wound care products as defined for this review. The full Medline search strategy was developed as follows: ("Biological Dressings" [Mesh] OR "Collagen" [Mesh] OR "Skin, Artificial" [Mesh] OR "Bandages" [Mesh] OR "Platelet-Derived Growth Factor" [Mesh]) AND ("Cost-Benefit Analysis" [Mesh] OR "economics" [Subheading]). Six studies could be retrieved by using this search algorithm. Table S1 provides further details on the searches [see Additional file 1].

Two reviewers independently undertook the assessment of study quality. Studies were assessed by an abbreviated synthesis of two recent quality checklists for health economic evaluations – the Consensus Health Economic Criteria (CHEC) list [3] and a checklist for quality assessment in decision-analytic models developed for health technology assessment by Philips et al. [4]. The CHEC-list established by Evers et al. is limited to systematic reviews that include full health economic evaluations based on clinical trials; that is, it is not suitable for economic evaluation studies based on decision-analytic modelling or scenario-analysis. Furthermore, the CHEC-list can be used only for systematic reviews which are based on full health economic evaluations and compare costs and outcomes of at least two interventions, and in which the costs and outcomes of those interventions are examined. Thus, key modelling issues were evaluated using the quality assessment tool for decision-analytic models established by Philips et al. This checklist consists of three key dimensions of quality: structure, data and consistency. The abbreviated synthesis of the two quality checklists described above consists of 23 items. Health economic evidence was considered good, if the health economic evaluations met the standard of transparency, if the underlying results were stable in the sensitivity analysis and if no unexplained inconsistencies between studies were observed. Table S2 displays the results of the study appraisal [see Additional file 2]. Characteristics of economic evaluations are summarised in table S3 providing further details on study methodology [see Additional file 3]. Methodological issues of economic evaluations are provided in table S4 [see Additional file 4].

## Results

Eleven health economic evaluations were included. Three biotechnology products were identified for which cell-derived wound care products for the treatment of chronic leg ulcers were economically assessed. All products are already available on the market: (1) Apligraf, a bilayered living human skin equivalent indicated for the treatment of diabetic foot and venous leg ulcers (five studies); (2) Dermagraft, a human fibroblast-derived dermal substitute, which is indicated only for use in the treatment of full-thickness diabetic foot ulcers of duration greater than 6 weeks that extend through the dermis, but without tendon, muscle, joint capsule, or bone exposure (one study); (3) REGRANEX Gel (becaplermin), a human platelet-derived growth factor for the treatment of deep neuropathic diabetic foot ulcers (five studies). Table 1 provides a brief overview of the products in the economic evaluations.

**Table 1:** Overview of human cell-derived wound care products for the management of chronic leg ulcers investigated by health economic evaluation

**Product**	**Description**	**Indications**	**Manufacturer**
Apligraf^®^	Bi-layered skin substitute: the epidermal layer is composed of human keratinocytes; the dermal layer is formed by human fibroblasts in a bovine type I collagen matrix	▪ Non-infected partial and full-thickness venous leg ulcers ▪ Full-thickness neuropathic diabetic foot ulcers	Organogenesis, US

Becaplermin (REGRANEX^® ^Gel)	Clear colourless to straw-coloured gel, which contains 0.01% of the active substance becaplermin	▪ Deep neuropathic diabetic foot ulcers	Systagenix Wound Management, US

Dermagraft^®^	Cryopreserved human fibroblast-derived dermal substitute composed of fibroblasts, extracellular matrix, and a bioabsorbable scaffold	▪ Full-thickness diabetic foot ulcers	Advanced BioHealing, US

### Establishment of effectiveness

Many studies were based on randomised controlled clinical trials. In three of the studies considered here [5-7], becaplermin efficacy was derived from a meta-analysis conducted by Smiell et al. [8] of four randomised studies including 922 patients with diabetic foot ulcers. Kantor and Margolis [9] took the becaplermin efficacy data from a review by Wieman [10] of four multicentre, randomised, placebo-controlled, parallel group studies. These four studies included a total of 922 patients. Sibbald et al. [11] used the multicentre double-blind placebo-controlled phase III trial of 382 patients by Wieman et al. [12]. In the Apligraf study by AÉTMIS [13], data were derived from the pivotal clinical trial by Falanga et al. [14] including 293 venous ulcer patients to calculate the primary outcome measure. Steinberg et al. [15] took effectiveness data from the randomised prospective trial by Veves et al. [16] to evaluate the cost-effectiveness of Apligraf in the treatment of diabetic foot ulcers. Finally, in the Dermagraft study by Segal and John [17], the US pivotal clinical trial by Naughton and colleagues [18] of 235 patients was used to assess the mean cost per ulcer healed.

An overview of the sources for effectiveness data used in the evaluations is given in table S5 [see Additional file 5]. Table 2 provides an overview of the studies and systematic reviews used for the assessment of effectiveness.

**Table 2:** Characteristics of the randomised clinical trials for establishing effectiveness

Wound care product	Country	Study period	Number of patients/study design	Initial ulcer size (cm^2^)	Mean age	Dressings	Ulcers healed (%/duration)	Reference
Apligraf	US	12 w + 3 m (follow-up)	112 (Apligraf), 96 (saline-moistened gauze), RCT	2.97 intervention group (IG), 2.83 control group (CG)	58 IG, 56 CG	Weekly application of Graftskin for a maximum of 4 weeks	56/12 w IG, 38/12 w CG	Veves et al. 2001

Apligraf	US	6 m + 6 m (follow-up)	146 (Apligraf plus compression), 129 (compression alone), RCT	1.33 IG, 1.05 CG	60.2 IG, 60.4 CG	Maximum of 5 in weeks 0–3, (IG), 1 in weeks 0–8 (CG)	63/6 m IG, 48.8/6 m CG	Falanga et al. 1998

Dermagraft	US	32 w	109 (Dermagraft plus conventional therapy, 126 (conventional therapy alone), RCT	≥ 1	Not stated	Weekly application of Dermagraft in weeks 0–7	38.5/12 w IG, 31.7/12 w CG	Naughton et al. 1997

Becaplermin	US	20 w	*Study 1: *57 P, 61 B (30 μg/g); Study 2: *; *Study 3: *68 GUC alone, 70 P, 34 B (100 μg/g); *Study 4: *122 GUC, 128 B (100 μg/g); *Total*: 190 GUC, 254 P, 193 B (30 μg/g), 285 B (100 μg/g), 478 B (all doses), review of 4 RCTs	*Study 1*: 7.2, *Study 2: 2.7*, *Study 3*: 2.2, *Study 4: 2.9*	*Study 1: 61*, *Studies 2, 3: 58*, *Study 4: 60*	Becaplermin or placebo gel was applied topically once daily and covered with saline-moistened gauze.	*Study 1*: 48/20 w B (30 μg/g), 25/20 w P; *Study 2*: *; *Study 3*: 44/20 w B (100 μg/g), 36/20 w P, 22/20 w GUC; *Study 4*: 36/20 w B (100 μg/g), 32/20 w GUC	Wieman 1998

Becaplermin	US	20 w	*, meta analysis of 4 RCTs	*Study 1: *9.0 P, 5.5 (30 μg/g); Study 2: *; *Study 3: *2.5 GUC, 2.2 P, 1.6 B (100 μg/g); *Study 4: *2.5 GUC, 3.2 B (100 μg/g)	*Study 1: *58 P, 63 (30 μg/g); Study 2: *; *Study 3: *60 GUC, 57 P, 59 B (100 μg/g); *Study 4: *60 GUC, 59 B (100 μg/g)	**	* for efficacy results of individual studies; combined analysis of treatment efficacy: 36/20 w (GUC/P), 42/20 B (30 μg/g), 50/20 B (100 μg/g)	Smiell et al. 1999

Becaplermin	US	20 w	127 P, 132 B (30 μg/g), 123 B (100 μg/g), RCT	2.8 P, 2.6 B (30 μg/g), 2.6 B (100 μg/g)	58 P, 58 B (30 μg/g), 57 B (100 μg/g)	**	50/20 w B (100 μg/g), 36/20 w B (30 μg/g), 35/20 w P	Wieman et al. 1998

*: See Wieman et al. 1998; **: See Wieman 1998; B: becaplermin; CG: control group; GUC: good ulcer care; IG: intervention group; m: months; P: placebo; RCT: randomised controlled trial; w: weeks.

### Establishment of cost-effectiveness

As the identified health economic evaluations were too heterogeneous and did not report adequate details for a meta-analysis of results, we performed a narrative synthesis of economic evidence including a description of all relevant information regarding study methods and cost-effectiveness results. In addition, in table 3, a summary table is provided presenting key information relating to type of ulcers, country, perspective, comparison of interventions, primary measure of health benefit and incremental or average cost-effectiveness ratios.

**Table 3:** Overview of health economic evaluations of cellular wound care products

**Wound care product**	**Source: author, country, year**	**Type of ulcers**	**Interventions**	**Perspective**	**Type of econ. eval**.	**Primary outcome measures/source of effectiveness evidence**	**Cost-effectiveness results (base case)**	**Sources of funding**	**Evidence**
Skin Substitutes									

Apligraf^®^^a^	AÉTMIS, CA, 2000	Venous leg	(1) Compression alone (2) Compression plus Apligraf^® ^simultaneously (3) Compression plus Apligraf^® ^for hard-to-heal ulcers	Societal/health care system	CEA^g^	Number of ulcer days averted/Falanga et al. 1998	The incremental cost per ulcer day averted of compression and Apligraf^® ^simultaneously used vs. compression alone was Can $26 (US $22)^d^, and Can $22 (US $18)^d ^when Apligraf^® ^was restricted to hard-to-heal ulcers. The price year was not reported.	Not stated	Limited^h, i^

	Harding et al., UK, 2000	Venous leg	(1) Saline gauze (2) Granuflex^®^^e^ (3) Apligraf^®^	Health care payer	CEA^g^	12-week healing rate/literature review	The cost per wound healed was £541 (US $828)^d ^for saline gauze, £341 (US $522)^d ^for Granuflex^®^, and £6,741 (US $10,323)^d ^for Apligraf. The price year was 1999.	ConvaTec	

	Kerstein et al., US, 2001	Venous leg	(1) Impregnated gauze (2) DuoDERM^®^^f^ (3) Apligraf^®^	Health care payer	CEA^g^	Number of persons healed/not healed after 12 weeks/literature review	The cost per patient healed was US $2,939 for impregnated gauze, US $1,873 for DuoDERM^®^, and US $15,053 for Apligraf^®^. The price year was 2000.	ConvaTec	

	Meaume/Gemmen, FR, 2002	Venous leg	(1) Saline gauze (2) DuoDERM^®^^f^ (3) Apligraf^®^	Health care payer	CEA^g^	Number of persons healed/not healed after 12 weeks/literature review	The cost per patient healed was £1,722 (US $1,832)^d ^for saline gauze, £1,018 (US $1,083)^d ^for DuoDERM^®^, and £ 15,920 (US $16,936)^d ^for Apligraf^®^. The price year was 1999/2000.	ConvaTec	

	Steinberg et al. US, 2002	Diabetic foot	(1) Saline-moistened gauze alone (2) Saline-moistened gauze plus Apligraf^®^	Health care payer	CEA	Number of ulcer-free months gained, number of amputations or resections avoided/Veves et al. 2001	The incremental cost of Apligraf^® ^vs. control per ulcer-free month gained was US $6,683, and US $86,226 when amputations or resections avoided were considered as benefit measures. The price year was 2000.	Novartis	

Dermagraft^®^^b^	Segal/John, AU, 2002	Diabetic foot	(1) Convent. management alone (2) Convent. management plus Dermagraft^®^	Health care payer	CEA^g^	Number of healed weeks/Naughton et al. 1997	The incremental cost per additional healed week of Dermagraft^® ^vs. control was A $383 (US $292)^d^. The price year was 2000.	Smith + Nephew Pty. Ltd.	Limited^h, i, j^

Growth Factors									

Becaplermin (Regranex^®^^c^)	Ghatnekar et al., UK, 2000	Diabetic foot	(1) Good wound care (GWC) alone (2) GWC plus becaplermin	Health care system	CEA^g^	Number of ulcer days averted/Smiell et al. 1999	Becaplermin plus GWC was found to be cost saving. The price year was not reported.	Not clearly stated	Good

	Ghatnekar et al., CH/FR/SE/UK, 2001	Diabetic foot	(1) GWC alone (2) GWC plus becaplermin	Health care system	CEA^g^	Number of ulcer-free months gained/Smiell et al. 1999	Becaplermin plus GWC was found to be cost saving in Sweden, Switzerland and the UK. The incremental cost per ulcer-free month gained of becaplermin over GWC alone was US $19 in France. The price year was 1999.	R.W. Johnson Pharmaceutical Research Institute	

	Kantor/Margolis, US, 2001	Diabetic foot	(1) Standard care at a primary care setting (SC) (2) Standard treatment at a specialised wound care centre (WCC) (3) Treatment with platelet releasate at a wound care centre (PR) (4) Becaplermin	Health care payer	CEA^g^	Percentage of ulcers healed after 20 weeks/Wieman 1998	The incremental cost per additional 1% of ulcers healed (most expensive vs. least expensive) was US $36.59 (SC vs. becaplermin), and US $70.86 (becaplermin vs. WCC). Becaplermin dominated PR. The price year was 1999.	Curative Health Services, National Institutes of Health Geriatric Epidemiology	

	Persson et al. SE, 2000	Diabetic foot	(1) GWC alone (2) GWC plus becaplermin	Health care system	CEA^g^	Number of ulcer-months avoided/Smiell et al. 1999	Becaplermin plus GWC was found to be cost saving. The price year was 1999.	R.W. Johnson Pharmaceutical Research Institute	

	Sibbald et al., CA, 2003	Diabetic foot	(1) Best clinical care (BCC) alone (2) BCC plus becaplermin	Societal	CEA^g^	Number of ulcer days averted/Wieman et al. 1998	The ICER was Can $6 (US $5)^d^. The costs were estimated in 1998 and updated to 2002 costs using the Canadian Consumer Price Index for Personal and Health Care.	Janssen-Ortho Inc.	

^a ^Apligraf^® ^is a registered trademark of Novartis. ^b ^Dermagraft^® ^is a registered trademark of Advanced BioHealing, Inc. ^c ^Regranex^® ^is a registered trademark of Systagenix Wound Management, Inc. ^d ^US $ converted by purchasing-power parity rates of the publication year. ^e ^Also known as DuoDERM^® ^in France and in the US. ^f ^DuoDERM^® ^is a registered trademark of Convatec. ^g^Cost-effectiveness model. ^h ^Inappropriate treatment of uncertainty.^i ^Small sample sizes.^j ^Only average cost-effectiveness ratios. AÉTMIS: Agence d'évaluation des technologies et des modes d'intervention en santé; AU: Australia; BCC: best clinical care; CA: Canada; CEA: cost-effectiveness-analysis; CH: Switzerland; DE: Germany; FR: France; GWC: good wound care; ICER: incremental cost-effectiveness ratio; PR: platelet releasate; SE: Sweden; UK: United Kingdom; US: United States.

### Apligraf^®^

In our literature search, we identified 5 health economic evaluations that assessed the cost-effectiveness of Apligraf^® ^in the management of venous leg or diabetic foot ulcers. All but one study were cost-effectiveness models and most economic evaluations were funded by manufactures/patentees of the products. Due to methodological weaknesses (i.e. only average cost-effectiveness ratios, small sample sizes, short time horizons, inadequate treatment of uncertainty), the quality of the evidence was considered limited.

An analytical prediction model was developed at AÉTMIS (2001) [13] by assembling information for the following treatment options of venous leg ulcer patients: compression alone, compression and Apligraf simultaneously, compression and Apligraf for hard-to-heal ulcers which are unresponsive to conventional therapy. To assess the number of ulcer days averted, which was used as the benefit measure in the economic analysis, data from the pivotal study by Falanga et al. (1998) [14] were obtained (further information on clinical trial characteristics are available in table 2). From both a health-care system perspective and a societal perspective the incremental cost for each ulcer day averted was calculated to amount to Can $26 when compression and Apligraf were used simultaneously, and Can $22 when compression and Apligraf were used for hard-to-heal ulcers.

Harding et al. (2000) [19] combined a summary of published studies and expert opinion to compare the cost-effectiveness of saline gauze, the hydrocolloid dressing Granuflex^® ^(UK trade name for DuoDERM^®^), and the human skin equivalent Apligraf for venous leg ulcer patients, over a 12-week treatment period. They identified 12 studies involving 843 ulcers, of which 205 were treated with saline gauze, 509 with Granuflex, and 278 with Apligraf. Due to heterogeneity across studies, only the percentage of ulcers healed was reported and used for the effectiveness estimate. The views of a European panel of four wound-care specialists on the issue of resource utilisation and costs were used to design protocols of care where data were not available from the literature. No summary measure of benefit was provided in the economic analysis. Cost-effectiveness was calculated from a health-care payer's perspective as the total medical cost of care of the cohort divided by the number of healed wounds, which yielded a cost per healed wound of £342 for Granuflex (nurse time £97, compression £121, dressing £124 and other £0), £541 for saline gauze (nurse time £327, compression £166, dressing £48 and other £0), and £6,741 for Apligraf (nurse time £70, compression £144, dressing £6,526 and other £1). An incremental cost-effectiveness ratio was not calculated.

Kerstein et al. (2001) [20] undertook a study in the United States, similar to that by Harding et al. [19], with a broader literature review to assess the cost-effectiveness of gauze dressings impregnated with saline, paraffin or zinc oxide, as compared with hydrocolloid dressings, and human skin construct (Apligraf) in venous leg ulcer patients. They included 18 studies and identified 223 patients on impregnated gauze dressings, 530 on hydrocolloid dressings, and 130 on Apligraf. The data on effectiveness were derived from a review of published studies. The benefit measure used in the economic analysis was the number of persons healed or not healed in a hypothetical managed-care plan with 100,000 covered lives. The average cost per patient healed was lowest with the hydrocolloid dressing DuoDERM (US $1,873), followed by impregnated gauze dressings at US $2,939, and then the human skin equivalent Apligraf at US $15,053 over a treatment period of 12 weeks.

Meaume and Gemmen (2002) [21] also used a similar methodology to that adopted by Harding et al. [19] to evaluate the cost-effectiveness of saline gauze, the hydrocolloid dressing DuoDERM (German trade name Varihesive^® ^and UK trade name Granuflex), and Apligraf in venous leg ulcer patients from a European and French perspective, respectively. The French perspective in this study was provided by a panel of five French wound-care experts. The European expert panel comprised four specialists from the UK, France, Germany, and Sweden. The evidence on effectiveness was derived from a literature review. The benefit measure used in the economic analysis was the number of patients healed at 6 and/or 12 weeks in a hypothetical cohort of 100,000. Although the treatment patterns are different between the United Kingdom and France, the pattern of cost-effectiveness per patient healed was consistent with the study conducted by Harding et al. [19] with the hydrocolloid dressing DuoDERM being most cost-effective at £2,763/1,018, followed by saline gauze at £1,436/1,722 and Apligraf at £11,396/15,920 from a European and a French perspective, respectively.

In the economic analysis by Steinberg et al. (2002) [15] the living skin equivalent Apligraf was compared to saline-moistened gauze to assess the cost-effectiveness of these two dressings in diabetic foot ulcer patients, on the basis of data from a six-month, multicentre, randomised trial [16]. In this trial, there were 112 patients in the intervention group and 96 controls. The benefit measures used in the economic analysis were the number of ulcer-free months gained and the number of amputations or resections avoided. In comparison to controls, patients in the living skin equivalent group had a higher average number of ulcer-free months (2.3 in the intervention group vs. 1.5 in the control group) and a lower average number of amputations or resections (5.4% in the intervention group vs. 12.5% in the control group). With a follow-up of six months, the incremental cost-effectiveness ratio of Apligraf over saline-moistened gauze was US $6,683 when the benefit measure was based on the number of ulcer-free months gained, and US $86,226 when amputations or resections avoided were considered.

### Dermagraft^®^

Segal and John (2002) [17] used observational case studies and a Markov model to evaluate the cost-effectiveness of Dermagraft in addition to conventional management in diabetic patients with neuropathic foot ulcers. The Markov model was used to assess average cost per ulcer healed, on the basis of effectiveness data from the pivotal clinical trial [18]. The benefit measures used in the model were the incremental healed weeks. On the basis of an observational case study of 27 hard-to-heal ulcers an alternative cost-effectiveness estimate was established, using a prospective analysis of resource use and cost for Dermagraft. A survey in the specialist clinic setting was used to assess the average cost of conventional management. On the basis of the Markov model, the average cost per ulcer healed was A $10,906 with conventional management and A $12,128 with Dermagraft as an additional treatment option. Using the results of the observational case studies, the average cost to treat an ulcer was A $12,500 prior to Dermagraft treatment and A $4,682 after starting Dermagraft treatment respectively.

### Becaplermin (REGRANEX^® ^Gel)

In our literature search, we found 5 economic evaluation studies that assessed the cost-effectiveness of becaplermin in the management of diabetic foot ulcers. All studies were cost-effectiveness models. Most of these cost-effectiveness analyses were funded by pharmaceutical companies or coauthored by their employees, and reported results that favoured the sponsor's product. The quality of the evidence was considered high.

The following three studies [5-7] used the same source of effectiveness evidence. However, country-specific patterns of resource usage and prices have a high impact on cost-effectiveness estimates. Therefore, to ensure that country-specific differences in treatment patterns and cost estimates are appropriately accounted for, the findings of these studies are reported here separately.

Persson et al. (2000) [5] used a Markov model to evaluate the cost-effectiveness of using becaplermin gel in combination with good wound care, as opposed to good wound care alone, to treat diabetic patients in Sweden who presented neuropathic, lower extremity ulcers. As part of a recent study on the cost of illness, a Markov model was developed [22]. With that as a starting point, the authors developed a more complete model of diabetic lower extremity ulcers. Data on resource usage were derived from a series of Swedish studies [23-26]. The becaplermin efficacy was taken from a combined analysis of four randomised studies [8]. The primary benefit measure used in the model was the number of ulcer-days averted. In comparison to controls, patients in the intervention group had a higher average number of ulcer-free months (4.22 in the intervention group vs. 3.41 in the control group) and a lower average number of amputations (5.91% in the intervention group vs. 6.50% in the control group). The average expected costs were US $12,078 for good wound care alone and US $11,708 for becaplermin.

In the economic analysis by Ghatnekar et al. (2000) [6] a similar Markov model to that developed by Persson et al. [5] was used to evaluate the cost-effectiveness in the UK of becaplermin gel as an adjunct therapy to good wound care in diabetic foot ulcer patients. Data on resource usage were taken primarily from a Swedish study [25]. To ensure the generalisability of the study results to the UK setting, they were reviewed by an expert panel of UK physicians. Data on becaplermin efficacy were drawn from the analysis by Persson et al [5]. The primary benefit measure used in the model was the number of ulcer-days averted. As in the analysis by Persson et al. [5] the average number of months spent in the healed state rose by 24% from 3.41 to 4.22 and the average number of amputations fell from 6.50% to 5.91% – a decline of 9%. The average expected costs over a 12-month time horizon were £10,880 for good wound care alone and £10,403 for treatment with becaplermin.

Ghatnekar et al. (2001) [7] used the same Markov model as the one developed by Persson et al. [5] to assess cost-effectiveness in four European countries (France, Sweden, Switzerland, UK) of becaplermin as an adjunct treatment option to good wound care alone in patients with diabetic foot ulcers. Another objective was to address the issue of generalisability by analysing the effect of different resource use patterns on the economics of managing diabetic foot ulcers. Data on effectiveness evidence for becaplermin were drawn from a pooled analysis of four randomised trials [8]. Resource data were primarily taken from a series of Swedish studies [23-25]. The primary benefit measure used in the model was the number of ulcer-months avoided. The expected number of months in the healed state was higher in the becaplermin group, 4.22 months vs. 3.41 months – an increase by 24%. Furthermore it was assumed that the average number of amputations would decline by 9%, from 6.50 to 5.91 per 100 patients. The average expected costs over a 12-month time horizon in the becaplermin group and the control group respectively were US $11,977/11,993 for France, US $12,168/11,783 for Sweden, US $14,112/13,832 for Switzerland, and US $17,601/17,133 for the UK.

Kantor and Margolis (2001) [9] used published literature and a database to assess the cost-effectiveness of various treatment options for diabetic neuropathic foot ulcers. The following treatment strategies were considered: standard care, standard treatment in a wound care centre, becaplermin, or platelet releasate. The becaplermin data were taken from a meta-analysis conducted by the authors on published studies of becaplermin gel 0.01% [10]. The measure of benefit used in the economic analysis was the percentage of ulcers healed. At 20 weeks of care the percentages of ulcers healed were 30.9% for standard care, 43% for becaplermin, 36.8% for platelet releasate, and 35.6% for treatment in a wound care centre. The incremental cost per patient of increasing by 1% the chance of healing at 20 weeks was US $36.6 for standard care vs. becaplermin, and US $70.86 for becaplermin vs. treatment in a specialised wound care centre. At this time, becaplermin was superior to platelet releasate, as a more effective and less costly treatment.

In the economic analysis by Sibbald et al. (2003) [11] a decision tree was used to assess the cost-effectiveness of becaplermin as an adjunct treatment option available to best clinical care in patients with nonhealing neuropathic diabetic foot ulcers. The effectiveness data were taken from a randomised controlled clinical trial of 251 diabetic patients [12]. The benefit measure used in the economic analysis was the number of ulcer-days averted. The number of ulcer-days per patient was 237 in the intervention group and 211 in the control group over a one year time horizon. Based on the model, the incremental cost-effectiveness ratio was Can $6 per ulcer-day averted.

While the cost-effectiveness of tissue engineered skin was considered poor in three studies [19-21], one study found it to be more cost-effective than compression alone [13]. This difference could be explained by the assumptions made in each analysis, most importantly the frequency of tissue engineered skin application in each model. Table 3 provides an overview of the economic evaluations. As described in the methods section, the last column is based on an appraisal of studies by two independent reviewers according to two published quality checklists.

### Comparison of the results to other studies

Ho et al. [2] included in their review three cost-effectiveness analyses of Apligraf [27-29], and one cost-effectiveness study of Dermagraft [1]. Studies assessed by that review were excluded from the present analysis to avoid 'double counting' of evidence, thus enhancing the efficient use of resources. A summary can be found in Table 4.

**Table 4:** Overview of economic evaluations included in Ho et al. 2005

Wound care product	Source: author, country, year	Type of ulcers	Interventions	Perspective	Type of economic evaluation	Primary outcome measures/source of effectiveness evidence	Cost-effectiveness results (base case)	Sources of funding	Evidence
Skin Substitutes									

Apligraf^®^	Redekop et al., NL, 2003	Diabetic foot	(1) GWC alone (2) GWC plus Apligraf^®^	Societal	CEA	Number of ulcer-free months gained and amputations avoided/Veves et al. 2001	Treatment with Apligraf (more effective and less costly) dominated over GWC alone.	Novartis	Limited
	
	Schonfeld et al., US, 2000	Venous leg	(1) Unna's boot (2) Apligraf^®^	Health care payer	CEA	Number of healed months and total % healed at 12 months/Falanga et al. 1998	Apligraf was the dominant strategy (more effective and less costly).	Novartis	
	
	Sibbald et al., CA, 2001	Venous leg	(1) 4-layer bandage system alone (2) 4-layer bandage system plus Apligraf^®^	Societal/Health care payer	CEA	Number of ulcer days averted/Falanga et al. 1998	Over a 3-month time horizon, the incremental cost per ulcer day averted with Apligraf plus 4-layer bandage system over 4-layer bandage system alone was Can $14 (US $12)* from both perspectives.	Novartis	

Dermagraft^®^	Allenet et al., FR, 2000	Diabetic foot	(1) Standard treatment (2) Dermagraft^®^	Societal	CEA	Number of additional ulcers healed/Naughton et al. 1997	The incremental cost per additional ulcer healed of Dermagraft^® ^over standard treatment was FF38,784 (US $41,260)*.	French Ministry of Health	Limited

*: US $ converted by purchasing-power parity rates of the publication year; CA: Canada, CEA: cost-effectiveness-analysis; FR: France, GWC: good wound care; NL: Netherlands; US: United States.

In the four health economic evaluations, univariate and multivariate sensitivity analyses respectively were conducted on probabilities and costs. The results of the sensitivity analyses showed that the models were generally robust to almost all variations in model inputs. The study results by Sibbald et al. [29] were sensitive to changes in the time loss from usual daily activities, in which case Apligraf plus four-layer bandage became the dominant treatment modality. In the analysis by Schonfeld et al. [28], when the cost of hospitalisation for patients in the Apligraf group was doubled, Apligraf was no longer the dominant strategy. In the economic evaluation by Redekop et al. [27], the incremental cost-effectiveness ratio was seen to be sensitive to the required number of applications of Apligraf, to amputation costs and differences in infection rate. For all three parameters the results of the incremental cost-effectiveness ratios ranged from cost-effective to cost-saving.

## Discussion

As no evaluation met all quality criteria, the evidence can in general be considered limited.

### Establishment of effectiveness

In none of the studies the clinical evidence was solely based on the 'gold standard' of randomised, clinical trials. Instead, cost-effectiveness was frequently modelled on the basis of observational data and, in some cases, supplemented by the author's assumptions and/or expert opinions.

All studies used condition-specific measures of benefit: percentage of ulcers healed [9,19-21], ulcer-days averted [6,11,13], additional healed weeks [17], ulcer-free months gained [5,7,15] and amputations avoided [15]. While these may represent standard effectiveness measures of wound care, these cost-effectiveness ratios do not allow for a meaningful comparison of cost-effectiveness across disease areas or a comparison with societal willingness to pay for a health gain. A more generic measure, for example the quality-adjusted life-years, would be desirable. Various studies considered health-related issues on overall quality of life in patients with venous leg and diabetic foot ulcers respectively [30-33], but our literature research identified no cost-utility analyses in this field. This may be due to a lack of studies that take into account quality of life in the health care interventions considered here. Only one study could be identified, in which quality of life with relation to the use of Apligraf was taken into account [34].

An important limitation of the publications considered in this review was the short time horizon. In three studies [19-21], the time horizon was twelve weeks, which gives rise to the following problem [20]: as a result of the short time horizon, the average healing cost is comparatively high, because dressings are applied in the first weeks of treatment. Thus, over a longer time horizon, the average treatment cost may be expected to become lower. In some studies, the time horizon was limited to that used in the primary studies. Two studies considered longer time periods in one-way sensitivity analyses [5,6]. The authors of these studies suggest that the cost-effectiveness may be more favourable over a longer time horizon.

In some studies, the results were reported as cost per wound healed. This does not consider additional factors, such as the length of time needed for the wound to heal, pain, or other complications which may arise during the healing process. It may be that a particular treatment has a higher cost per wound healed compared to other treatments, but helps heal a larger number of wounds.

### Establishment of cost-effectiveness

None of the identified products fully met all quality criteria and frequently deviated from current standards of health economic evaluation. For example, Harding et al. [19], Kerstein et al. [20], and Meaume and Gemmen [21] did not calculate incremental cost-effectiveness ratios but only reported average cost-effectiveness. In many cases, the perspective was not stated clearly or substantiated. Also, quantities of consumed resources or details on discounting were often not given. Probabilistic sensitivity analysis was conducted in none of the eleven health economic evaluations. Most of the economic evaluations relied on one-way sensitivity analyses.

The categories of costs included in the evaluations differed substantially between studies. Most studies considered only direct costs (e.g. outpatient clinic visits, hospital days, physician office visits, homecare, laboratory and diagnostic tests, debridement, dressing materials, orthopaedic appliances, costs of amputations). Indirect costs were included in two evaluations only [11,13]. Intangible costs were not evaluated. In many studies, a detailed breakdown of the costs was not provided, the price year was not reported, and the unit costs were not presented separately from the quantities of resources used. Health resource use was often estimated on the basis of expert opinions.

The transferability of clinical and economic evidence is an important issue in health economic evaluation [35]. Some authors noted that a limitation of their studies was the lack of available data. In most evaluations, effectiveness data came from clinical trials conducted in the USA, but the application of US data to European countries could be controversial. Furthermore, the number of applications of skin substitutes may vary substantially between settings. The issue of generalisability of study results to other settings was extensively addressed only by one study [7], in which four different European countries were considered. The study by Ghatnekar et al. (2001) revealed substantial differences in treatment patterns and in the nature of cost-savings across countries, owing to the differences in the health care systems. In this context, reimbursement systems, relative prices, and treatment practices are important issues that vary from country to country [5]. In general, decision-makers in other health care systems should investigate the cost-effectiveness of tissue-engineered products in their own setting.

Discounting was of low importance since all costs were incurred within a period of less than a year. However, while many guidelines recommend equal discounting for costs and effects, discounting of costs only was performed in two studies [5,6].

### Limitations of the present review

First of all, it must be stated that the choice of dressings studied was dictated by the available fully published health economic evaluations. Currently, only few chronic leg ulcer treatment modalities provide sufficient details from which to draw conclusions concerning cost-effectiveness. Furthermore, only studies in English, French or German could be included. The economic analyses were appraised by a checklist on methodological quality and the studies considered were rated only on the basis of widely applicable criteria known to the authors. However, good health economic evaluations are more than the sum of their parts [36]. Even though the study appraisal was based on the consensus of two independent reviewers, it is nonetheless restricted by those reviewers' subjectivity.

## Conclusion

Cell-derived wound care products in addition to standard care generate very high costs. This is a consequence of the attempt to increase the number of ulcers healed or ulcer-free time. The economic evidence suggests that despite their high initial costs, tissue-engineered wound care products may be cost-effective or even cost-saving if their use is restricted to such ulcers that are unresponsive to healing. This is because the initial costs of novel biotechnology products may be offset by shorter treatment periods (i.e., higher healing rates and faster healing), fewer complications (i.e. infection and gangrene that carry a higher risk of amputation) and fewer inpatient episodes.

Due to the limitations of the studies included in this review, future work should focus on well-designed studies, using appropriate methods for randomisation. Furthermore, prospective studies should be conducted to corroborate current study results. As the cost-effectiveness of these treatment modalities may improve over time [29], future health economic analyses should explore a longer time horizon. Further research should also be carried out to obtain better estimates of the clinical benefits, since the findings of most studies were shown to be sensitive to these parameters, and further investigations into the effectiveness of the various treatments in routine clinical practice and patient-related outcomes (such as health-related quality of life) are needed to complement the existing data on cost-effectiveness and help reimbursement agencies make informed coverage decisions.

## Competing interests

The authors declare that they have no competing interests.

## Authors' contributions

AL conducted the searches and the review of clinical studies, participated in study appraisal, and took the lead in writing the manuscript. WR initiated the project, developed the concept, provided methodological supervision throughout the study and contributed to writing the manuscript. Both authors read and approved the final manuscript.

## Pre-publication history

The pre-publication history for this paper can be accessed here:



## Supplementary Material

Additional file 1 **systematic literature search (updated 07/25/2008)**. Additional file 1 provides further details on the searches. Click here for file

Additional file 2 **results of study appraisal**. Additional file 2 displays the results of the study appraisal Click here for file

Additional file 3 **characteristics of economic evaluations (n = 11)**. Additional file 3 provides further details on study methodology. Click here for file

Additional file 4 **methodological issues of economic evaluations**. Additional file 4 also provides further details on study methodology. Click here for file

Additional file 5 **source of effectiveness data**. Additional file 5 provides an overview of the sources for effectiveness data used in the evaluations. Click here for file
