# Supplementary material for: Systematic review of economic evaluations of human cell-derived wound care products for the treatment of venous leg and diabetic foot ulcers
Source: BMC Health Serv Res. 2009 Jul 10;9:115. doi: 10.1186/1472-6963-9-115 (PMC2716319; doi:10.1186/1472-6963-9-115)
Supplement: Additional file 1 — systematic literature search (updated 07/25/2008). Additional file 1 provides further details on the searches. [file 1472-6963-9-115-S1.doc]

## Table S1: systematic literature search (updated 07/25/2008)

| **Data base** | **Search** | **Queries** | **Hits** |
| --- | --- | --- | --- |
| *BIOSIS Previews* | #1 | (“biological dressings” OR “collagen” OR “artificial skin” OR “bandages” OR “platelet-derived growth factor”) | 263,480 |
|  | #2 | (apligraf OR graftskin OR dermagraft OR regranex OR becaplermin) | 145 |
|  | #3 | ((LA:PREV = ENGLISH) or (LA:PREV = FRENCH) or (LA:PREV = GERMAN)) | 13,877,568 |
|  | #4 | ((DT:PREV = EDITORIAL) or (DT:PREV = LETTER)) | 144,402 |
|  | #5 | (economic* OR cost*) | 620,857 |
|  | #6 | #1 OR #2 | 263,583 |
|  | #7 | #1 AND #2 AND #3 NOT #4 | 42 |
|  | #8 | (#1 OR #2) AND #3 AND #5 NOT #4 | 2,660 |
| *CRD databases (DARE, NHS EED, HTA data base)* | #1 | (“biological dressings” OR “collagen” OR “artificial skin” OR “bandages” OR “platelet-derived growth factor”) | 239 |
|  | #2 | (apligraf OR graftskin OR dermagraft OR regranex OR becaplermin) | 23 |
|  | #3 | (French:la OR German:la OR English:la) | 35,553 |
|  | #4 | (economic* OR cost*) | 2,801 |
|  | #5 | 1 OR 2 | 249 |
|  | #6 | 1 AND 2 AND 3 AND 4 | 12 |
|  | #7 | 3 AND 4 AND 5 | 163 |
| *Co­chrane Library* | #1 | (“biological dressings” OR “collagen” OR “artificial skin” OR “bandages” OR “platelet-derived growth factor”) | 4,133 |
|  | #2 | (apligraf OR graftskin OR dermagraft OR regranex OR becaplermin) | 97 |
|  | #3 |  |  |
|  | #4 | (editorial):pt or (letter):pt | 4,928 |
|  | #5 | (economic* OR cost*) | 53,094 |
|  | #6 | #1 OR #2 | 4,186 |
|  | #7 | #1 AND #2 NOT 4 | 44 |
|  | #8 | (#1 OR #2) AND #5 NOT #4 | 539 |
| *EconLit* | #1 | (“biological dressings” OR “collagen” OR “artificial skin” OR “bandages” OR “platelet-derived growth factor”) | 2 |
|  | #2 | (apligraf OR graftskin OR dermagraft OR regranex OR becaplermin) | 1 |
|  | #3 | LA English or LA German or LA French | 860,429 |
|  | #5 | (economic* OR cost*) | 508,906 |
|  | #6 | 1 OR 2 | 3 |
| *Embase* | #1 | (“biological dressings” OR “collagen” OR “artificial skin” OR “bandages” OR “platelet-derived growth factor”) | 85,888 |
|  | #2 | (apligraf OR graftskin OR dermagraft OR regranex OR becaplermin) | 565 |
|  | #3 | (LA:EMBV = ENGLISH OR LA:EMBV = FRENCH OR LA:EMBV = GERMAN) | 8,127,936 |
|  | #4 | (DT:EMBV = EDITORIAL OR DT:EMBV = LETTER) | 593,361 |
|  | #5 | (economic* OR cost*) | 333,549 |
|  | #6 | #1 OR #2 | 86,268 |
|  | #7 | #1 AND #2 AND #3 NOT #4 | 169 |
|  | #8 | (#1 OR #2) AND #3 AND #5 NOT #4 | 1,237 |
| *Medline* | #1 | (“biological dressings” OR “collagen” OR “artificial skin” OR “bandages” OR “platelet-derived growth factor”) | 144,083 |
|  | #2 | (apligraf OR graftskin OR dermagraft OR regranex OR becaplermin) | 218 |
|  | #3 | LA=“French” OR LA=“German” OR LA=“English” | 14,107,531 |
|  | #4 | (DT=“letter”? OR DT=“comment”?) OR DT=“editor”? | 922,323 |
|  | #5 | (economic* OR cost*) | 501,067 |
|  | #6 | 1 OR 2 | 144,134 |
|  | #7 | 1 AND 2 AND 3 NOT 4 | 153 |
|  | #8 | 3 AND 5 AND 6 NOT 4 | 1,547 |
| *Web of Science* | #1 | (“biological dressings” OR “collagen” OR “artificial skin” OR “bandages” OR “platelet-derived growth factor”) | >100000 |
|  | #2 | (apligraf OR graftskin OR dermagraft OR regranex OR becaplermin) | 257 |
|  | #3 | (economic* OR cost*) | >100000 |
|  | #4 | #1 OR #2 | >100000 |
|  | #5 | Topic=((“biological dressings” OR “collagen” OR “artificial skin” OR “bandages” OR “platelet-derived growth factor”)) AND Topic=((apligraf OR graftskin OR dermagraft OR regranex OR becaplermin)) AND Language=(English OR French OR German) NOT Document Type=(Editorial Material OR Letter) | 88 |
|  | #6 | Topic=((“biological dressings” OR “collagen” OR “artificial skin” OR “bandages” OR “platelet-derived growth factor”)) AND Topic=((apligraf OR graftskin OR dermagraft OR regranex OR becaplermin)) AND Topic=((economic* OR cost*)) AND Language=(English OR French OR German) NOT Document Type=(Editorial Material OR Letter) | 8 |
