# Supplementary material for: Systematic review of economic evaluations of human cell-derived wound care products for the treatment of venous leg and diabetic foot ulcers
Source: BMC Health Serv Res. 2009 Jul 10;9:115. doi: 10.1186/1472-6963-9-115 (PMC2716319; doi:10.1186/1472-6963-9-115)
Supplement: Additional file 2 — results of study appraisal. Additional file 2 displays the results of the study appraisal [file 1472-6963-9-115-S2.doc]

## Table S2: results of study appraisal

|  | **Apligraf (Graftskin)** | | | | | **Dermagraft** | **REGRANEX Gel (Becaplermin)** | | | | |  |
| --- | --- | --- | --- | --- | --- | --- | --- | --- | --- | --- | --- | --- |
| Item / Source  ** Applied only if Model-Based Economic Evaluation;*  ° Applied only if Trial-Based Econ. Eval. | AÉTMIS 2000 | Harding et al. 2000 | Kerstein et al. 2001 | Meaume, Gemmen 2002 | Steinberg et al. 2002 | Segal, John 2002 | Ghatnekar et al. 2000 | Ghatnekar et al. 2001 | Kantor, Margolis 2001 | Persson et al. 2000 | Sibbald et al. 2003 | Description of the assigned scores (0/0.5/1) |
| **Research Question/Perspective** |  |  |  |  |  |  |  |  |  |  |  |  |
| Is a well-defined research question posed in answerable form? | 1 | 1 | 0.5 | 1 | 0.5 | 1 | 1 | 1 | 0.5 | 1 | 1 | Clear identification of alternatives and study population: 1; less clear: 0.5; not clear: 0 |
| Is the chosen perspective appropriate? | 1 | 0.5 | 0.5 | 0.5 | 0.5 | 0.5 | 0.5 | 1 | 0.5 | 0 | 1 | Societal perspective or justification of a narrower perspective: 1; narrower perspective without justification: 0.5; not stated: 0 |
| **Intervention(s)** |  |  |  |  |  |  |  |  |  |  |  |  |
| Are competing alternatives clearly described? | 1 | 0.5 | 0.5 | 0.5 | 0.5 | 0.5 | 0.5 | 1 | 0.5 | 0.5 | 1 | Detailed description (e.g. objective/intensity/duration/frequency): 1; less detailed: 0.5; not detailed: 0 |
| *Is the study population clearly described?** | n.a. | n.a. | 1 | n.a. | 1 | 0.5 | 0 | 0.5 | 0 | 0.5 | 0.5 | Population is based on different studies (e.g. literature review): n.a.; detailed entry and eligibility criteria: 1; less detailed: 0.5; not detailed: 0 |
| Is the chosen time horizon appropriate so that relevant costs and consequences can be included? | 0 | 1 | 1 | 1 | 0.5 | 1 | 1 | 1 | 0 | 1 | 1 | Explicitly stated: 1; not explicitly stated, but obvious: 0.5; not stated: 0; |
| Data identification: are the data identification methods transparent and appropriate, given the objectives of the model? | 1 | 1 | 1 | 1 | 0 | 0.5 | 0 | 0.5 | 0.5 | 0 | 0.5 | Detailed description of literature search and its results: 1; less detailed: 0.5; not detailed: 0 |
| Data selection: where choices have been made between data sources, are these justified appropriately? | 1 | 1 | 1 | 1 | 0 | 0.5 | 0 | 0.5 | 0 | 0.5 | 0.5 | Detailed description of choice criteria of input parameters: 1; less detailed: 0.5; not detailed: 0 |
| Data transparency: have all data incorporated into the model been described and referenced in sufficient detail? | 1 | 0.5 | 1 | 0.5 | 0 | 0.5 | 0.5 | 0.5 | 0.5 | 0.5 | 1 | Detailed description of data and their sources: 1; less detailed: 0.5; not detailed: 0 |
| **Costs** |  |  |  |  |  |  |  |  |  |  |  |  |
| Are all important and relevant costs for each alternative identified? | 1 | 1 | 0.5 | 1 | 0.5 | 1 | 0.5 | 1 | 1 | 1 | 1 | Full identification of cost items (e.g. dressing materials, nursing time, hospitalisation for diabetic foot ulcers): 1; if important and relevant costs were omitted: 0.5; only aggregated total costs: 0 |
| Are all costs measured appropriately in physical units? | 0 | 0.5 | 0.5 | 0.5 | 0 | 1 | 0 | 1 | 0.5 | 0.5 | 1 | Separate reporting of quantities and unit costs: 1; separate reporting for certain, but not all, cost items: 0.5; no separate reporting: 0 |
| Are costs valued appropriately? | 0.5 | 0.5 | 1 | 0.5 | 0.5 | 1 | 0.5 | 0.5 | 1 | 0.5 | 1 | Clear statement of sources and reference year: 1; sources of cost prices and/or reference year unclear: 0.5; neither reference year nor sources reported: 0 |

| **Outcomes** |  |  |  |  |  |  |  |  |  |  |  |  |
| --- | --- | --- | --- | --- | --- | --- | --- | --- | --- | --- | --- | --- |
| Are all important and relevant outcomes for each alternative identified? | 1 | 0.5 | 0.5 | 0.5 | 1 | 1 | 1 | 1 | 0.5 | 1 | 1 | Outcome: ulcer-day(s)/week(s)/month(s) avoided: 1; healing rate: 0.5 |
| *Are all outcomes measured appropriately?** | *1* | *0.5* | *0.5* | *0.5* | *0.5* | *0.5* | *n.a.* | *n.a.* | *n.a.* | *n.a.* | *0.5* | *Model-based economic evaluation: n.a.; clear statement of instruments: 1; less clear: 0.5; not clear/stated: 0* |
| If relative treatment effects have been derived from trial data, have they been synthesised using recognised meta-analytic techniques?° | 1 | 1 | 1 | 1 | n.a. | n.a. | n.a. | n.a. | 0.5 | n.a. | n.a. | Trial-based economic evaluation (TBEE)/only one study on which the model is based: n.a.; clear statement of data analysis/synthesis: 1; unclear statement: 0.5; not stated: 0 |
| Is the structure of the model consistent with a coherent theory of the health condition under evaluation? | 1 | 0 | 0 | 0 | 0 | 1 | 1 | 1 | 0 | 1 | 1 | TBEE: n.a.; model consistent with natural history of the disease (e.g. Markov model): 1; model inconsistent with natural history of the disease (e.g. decision tree): 0.5; model unclear: 0 |
| Are structural assumptions and model type transparent and justified? | 1 | 1 | 1 | 1 | 0 | 0.5 | 1 | 1 | 0 | 1 | 1 | TBEE: n.a.; clear structure of the model: 1; assumptions not transparent/justified: 0.5; assumptions not stated: 0 |
| Are the structural assumptions and model type reasonable, given the overall objective, perspective and scope of the model? | 0.5 | 1 | 1 | 1 | 0 | 0.5 | 0.5 | 1 | 0 | 1 | 1 | TBEE: n.a.; model including its description reasonable: 1; less reasonable: 0.5; another model would be the better option: 0 |
| **Analysis** |  |  |  |  |  |  |  |  |  |  |  |  |
| Is an incremental analysis of costs and outcomes of alternatives performed? | 1 | 0 | 0 | 0 | 1 | 0.5 | n.a. | 1 | 1 | n.a. | 1 | Cost-saving and higher outcomes: n.a.; ICER transparent: 1; less transparent/unclear: 0.5; no ICER: 0 |
| Are all future costs and outcomes discounted appropriately? | 0 | n.a. | n.a. | n.a. | n.a. | n.a. | 0.5 | n.a. | n.a. | 0.5 | n.a. | Short time horizon (**≤** 12 months): n.a.; discounting of costs and effects: 1; only costs/not motivated discount rate: 0.5; discount rate not stated: 0 |
| Are all important variables, whose values are uncertain, appropriately subjected to sensitivity analysis? | 1 | 0 | 0 | 0 | 1 | 0 | 0 | 0.5 | 0.5 | 1 | 0.5 | TBEE: n.a.; justification of the range used in the sensitivity analysis (SA): 1; transparent SA without justification: 0.5; no sensitivity analysis of important parameters: 0 |
| Do the conclusions follow from the data reported? | 1 | 1 | 1 | 1 | 1 | 1 | 1 | 1 | 1 | 1 | 1 | Conclusions justified: 1; less justified: 0.5; not justified: 0 |
| *Does the study discuss the generalisability of the results to other settings and patient/client groups?** | *0.5* | *0* | *0* | *0.5* | *0.5* | *0* | *0* | *1* | *0.5* | *1* | *0.5* | *Detailed discussion: 1; discussion short/incomplete/indirect: 0.5; no discussion of generalisability: 0* |
| Does the article indicate whether there is a potential conflict of interest where  study researcher(s) and funder(s) are concerned? | 0.5 | 0.5 | 0 | 0.5 | 0.5 | 0.5 | 0 | 0.5 | 1 | 0.5 | 0 | Full transparency: 1; limited transparency: 0.5; no transparency: 0 |
| **Mean Score** | **0.77** | **0.62** | **0.61** | **0.64** | **0.45** | **0.64** | **0.48** | **0.88** | **0.48** | **0.70** | **0.81** |  |
