# Supplementary material for: Systematic review of economic evaluations of human cell-derived wound care products for the treatment of venous leg and diabetic foot ulcers
Source: BMC Health Serv Res. 2009 Jul 10;9:115. doi: 10.1186/1472-6963-9-115 (PMC2716319; doi:10.1186/1472-6963-9-115)
Supplement: Additional file 3 — characteristics of economic evaluations (n = 11). Additional file 3 provides further details on study methodology. [file 1472-6963-9-115-S3.doc]

## Table S3: characteristics of economic evaluations (n=11)

| Article characteristics | | Number |
| --- | --- | --- |
| Year of publication | 2000 | 4 |
|  | 2001 | 3 |
|  | 2002 | 3 |
|  | 2003 | 1 |
| Country of study | Australia | 1 |
|  | Canada | 2 |
|  | France | 1 |
|  | Sweden | 1 |
|  | United Kingdom | 2 |
|  | United States | 3 |
|  | Other | 1 |
| Wound care product | Apligraf | 5 |
|  | Becaplermin | 5 |
|  | Dermagraft | 1 |
| Ulcer site | Diabetic Foot | 7 |
|  | Venous Leg | 4 |
| Journal | British Journal of Nursing | 1 |
|  | Dermatologic Surgery | 1 |
|  | Disease Management & Health Outcomes | 1 |
|  | Journal of Drug Assessment | 1 |
|  | Journal of Wound Care | 1 |
|  | Ostomy Wound Management | 1 |
|  | Pharmacoeconomics | 1 |
|  | Primary Intention | 1 |
|  | Value in Health | 1 |
|  | Wounds | 1 |
