# Supplementary material for: Systematic review of economic evaluations of human cell-derived wound care products for the treatment of venous leg and diabetic foot ulcers
Source: BMC Health Serv Res. 2009 Jul 10;9:115. doi: 10.1186/1472-6963-9-115 (PMC2716319; doi:10.1186/1472-6963-9-115)
Supplement: Additional file 4 — methodological issues of economic evaluations. Additional file 4 also provides further details on study methodology. [file 1472-6963-9-115-S4.doc]

## Table S4: methodological issues of economic evaluations

| Methodological issues | | Number |
| --- | --- | --- |
| Perspective* | Health Care Payer | 6 |
|  | Health Care System | 4 |
|  | Societal | 2 |
| Time horizon* | 12 weeks | 3 |
|  | 20 weeks | 1 |
|  | 12 months | 6 |
| Discounting* | Costs | 2 |
|  | Outcomes | 0 |
|  | Both | 0 |
| Cost-effectiveness models* | Decision Tree | 2 |
|  | Markov Model | 4 |
| One-way sensitivity analyses* | Relating to costs (e.g. number of Apligraf/Dermagraft units, duration of 1 becaplermin tube, cost of time loss for unpaid activities) | 12 |
|  | Relating to efficacy/effectiveness (e.g. becaplermin/good wound care efficacy, effectiveness of Apligraf/compression therapy/platelet releasate/standard care) | 13 |
|  | Relating to other methodological issues (e.g. time horizon) | 2 |
|  | Relating to study population characteristics (e.g. baseline ulcer duration) | 2 |

*Does not add up to 11 because more than one method, or none, may have been used in the same study.
