# Supplementary material for: Systematic review of economic evaluations of human cell-derived wound care products for the treatment of venous leg and diabetic foot ulcers
Source: BMC Health Serv Res. 2009 Jul 10;9:115. doi: 10.1186/1472-6963-9-115 (PMC2716319; doi:10.1186/1472-6963-9-115)
Supplement: Additional file 5 — source of effectiveness data. Additional file 5 provides an overview of the sources for effectiveness data used in the evaluations. [file 1472-6963-9-115-S5.doc]

## Table S5: source of effectiveness data

| **Wound care product** | **Reference of cost-effectiveness analysis** | **Associated clinical trial** |
| --- | --- | --- |
| Apligraf | AÉTMIS 2000 | Falanga et al. 1998 |
|  | Harding et al. 2000; Kerstein et al. 2001; Meaume, Gemmen 2000 | Falanga et al. 1998/Apligraf (Graftskin) [product package insert] |
|  | Steinberg et al. 2002 | Veves et al. 2001 |
| Dermagraft | Segal, John 2002 | Naughton et al. 1997 |
| Becaplermin | Ghatnekar et al. 2000; Ghatnekar et al. 2001; Persson et al. 2000 | Smiell et al. 1999 |
|  | Kantor, Margolis 2001 | Wieman 1998 |
|  | Sibbald et al. 2003 | Wieman et al. 1998 |
